# Workload and GP burnout: a survey and register-based study in Danish general practice

**DOI:** 10.3399/BJGPO.2023.0077

**Published:** 2024-02-28

**Authors:** Astrid Ibsen Bruun, Peter Vedsted, Henrik Schou Pedersen, Anette Fischer Pedersen

**Affiliations:** 1 Research Unit for General Practice, Aarhus, Denmark; 2 Department of Public Health, Aarhus University, Aarhus, Denmark; 3 Department of Clinical Medicine, Research Clinic for Innovative Patient Pathways, Silkeborg Hospital, Aarhus University, Aarhus, Denmark; 4 Department of Clinical Medicine, Aarhus University, Aarhus, Denmark

**Keywords:** general practitioners, workload, professional burnout, family practice, Denmark

## Abstract

**Background:**

Burnout is common among GPs. Previous studies have indicated an association between high workload and burnout among doctors.

**Aim:**

To assess the risk of burnout among single-handed GPs in Denmark in relation to self-reported and register-based workload.

**Design & setting:**

Questionnaire data from 312 Danish single-handed GPs and register data on their patients and provided services.

**Method:**

Burnout was measured using the Maslach Burnout Inventory-Human Services Survey (MBI-HSS). A composite burnout score of quartile points was calculated. The questionnaire provided information on working hours. Register data included number of services and patient list size. Association between composite burnout score and workload was estimated with binomial regression analyses adjusting for the GP’s age and sex, and social deprivation score of their patient lists.

**Results:**

Working >5 days a week in practice increased the risk of a high burnout score (adjusted risk ratio [RR] = 2.34, 95% confidence interval [CI] = 1.62 to 3.37). Spending >7.5 hours a day on patient-related tasks increased the risk of a high burnout score. The highest score was among GPs spending 8.5–9.5 hours a day on patient-related tasks (adjusted RR = 2.01, 95% CI = 0.90 to 4.51), although not statistically significant. There was no association between number of services and risk of burnout.

**Conclusion:**

Working 5 days a week in practice significantly increased the risk of burnout in Danish single-handed GPs. Spending >7.5 hours a day on patient-related tasks tended to increase the risk. We found no association between a high number of services and increased risk of burnout.

## How this fits in

Growing workload and GP burnout are increasing problems in primary care and studies have linked these aspects. Using Danish national registers and questionnaire data from GPs, we found that it was the duration of work rather than the workload itself, which was associated with the risk of developing burnout.

## Introduction

In spite of a high job satisfaction among GPs in Denmark, more than one-third have shown moderate or severe levels of work-related burnout, with increasing numbers over the past 20 years.^1^ These worrying statistics are also seen internationally.^1–4^ Meanwhile, patient demands, medical needs, and workload have increased.^5,6^ Some studies have found an association between burnout and workload based on subjective^7,8^ as well as objective measures of workload,^3,9–12^ while others have not.^13,14^ However, the risk of information bias is high, since most studies have relied predominantly on self-reported workload.^12,15^

The burnout syndrome is a response to prolonged emotional and interpersonal stressors on the job.^7,16^ Healthcare professionals are considered at high risk.^2–4,12,17,18^ Burnout is associated with musculoskeletal pain,^19^ depression,^15^ insomnia, alcohol and drug use, and suicidal ideation.^12,16,20,21^ It can hamper optimal patient treatment causing higher risk of medical errors, incautious prescribing behaviour, dysfunctional interactions between colleagues, and lowered cost-efficiency in health care.^12,15,17,20–26^ In contrast to this, doctors who experience high levels of occupational wellbeing are more likely to deliver high-quality health care.^23,27^ A resilient primary care is crucial to the healthcare system, preventing overcrowding in emergency departments and unnecessary referrals.^28,29^ Reducing burnout could therefore be a quality-improving initiative.^30^

The aim of this study was to analyse the risk of burnout in relation to objective measures of workload in Danish GPs in a combined questionnaire survey and register-based study. Our hypothesis was that a higher workload would be related to a higher risk of burnout.

## Method

### Setting

In Denmark, general practice is the primary access point to the publicly tax-funded healthcare system. Nearly all citizens are listed with a general practice, which they must contact for medical advice. GPs have an average of 1600 listed patients. Most access to other specialists and hospitals requires referral from a GP.^31^ According to a national contract, GPs must provide medical care all weekdays from 8 am to 4 pm. Many GPs are obliged to participate in out-of-hours (OOH) primary care. All GPs are independent contractors with the regional health authorities and responsible for administration of their practice. Remuneration consists of a mix between capitation and fee-for-service.

### Data collection

**Figure 1.**
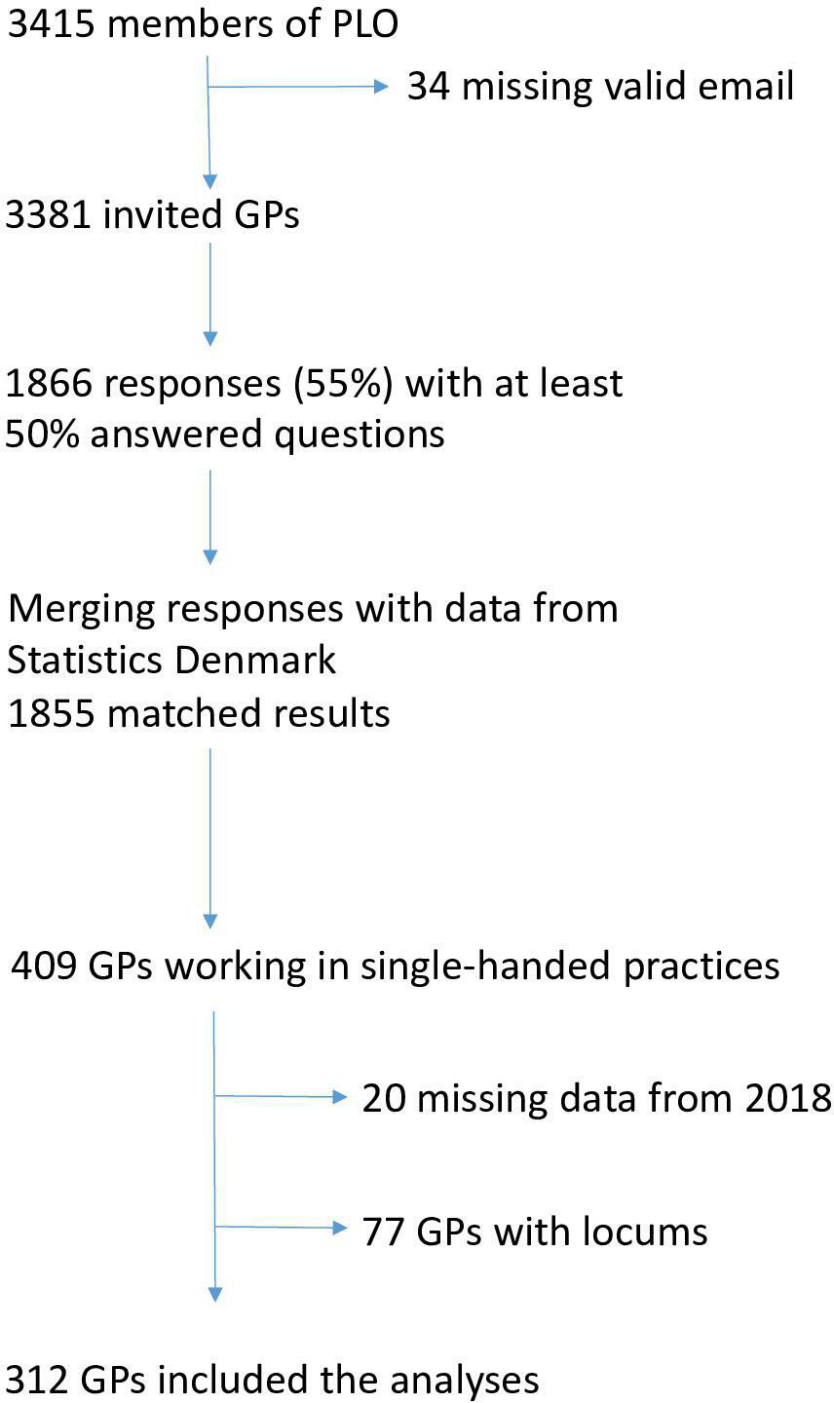
The inclusion of the 312 GPs in the analyses. PLO = Danish General Practitioners Organisation

This study is based on a national questionnaire survey on Danish GPs’ mental wellbeing and job satisfaction,^1,32^ which took place in November 2019. All 3381 GPs listed with a valid email address at the Danish General Practitioners Organisation (PLO) received an email with a personal link to an electronic questionnaire. In total, 1866 GPs returned the questionnaire (55.5%). To ensure accurate linkage of patients and workload with a single doctor, we only included GPs in single-handed practices for our analyses (409 GPs). For the same reason, we excluded GPs who had employed locums (77 GPs). Register data from 2019 was linked anonymously to the questionnaire data by provider number. To ensure that all included GPs had been active an entire year beginning on 1 January 2019, we checked whether the GPs had data on services registered in 2018. We excluded 20 GPs without any services registered in 2018, since we could not know when in 2019 they had become active in their practice. In total, 312 single-handed GPs were included in the analyses (Figure 1).

### Survey data

#### Composite burnout score

We used the Maslach Burnout Inventory–Human Services Survey (MBI–HSS) to assess burnout. This internationally used and validated self-assessment tool is considered the gold standard for assessment of the burnout syndrome.^7,20^ MBI–HSS is a 22-item questionnaire consisting of three subscales: emotional exhaustion, depersonalisation, and personal accomplishment. In line with previous studies,^32–34^ we categorised each subscale according to quartiles of the subscale sum scores (reversed for personal accomplishment) and combined these to a composite score. Thus, we assigned one point for subscale scores in the first quartile, two for the second, and so forth. The points were added up forming a composite burnout score ranging from 3–12, with a cut-off categorising a score from 9–12 as high degree of burnout.^34^

The following variables were derived from the questionnaire:

#### Patient-related working hours per day in practice

Time spent on patient-related tasks, including face-to-face consultations, telephone consultations, email correspondences, and other patient-related work including breaks.

#### Workdays in practice per week

Number of days in practice on a normal week.

### Register data

We obtained register-based data on workload from the Danish National Health Service Register for 2019. This register contains electronically collected data on all services provided in general practice for remuneration purposes.^35^

#### Number of listed patients

Number of listed patients as of 1 January 2019 was available from the Patient List Register, an administrative database containing information on practice affiliation at any given time.^36^

#### The Danish Deprivation Index (DADI) score

The Danish Deprivation Index (DADI) score,^37^ developed for use in general practice, is calculated by use of information on socioeconomic characteristics (education, income, employment status, household, and immigration status) of each patient, listed with a specific practice. DADI score is described in detail elsewhere.^37^ The index was defined from 1 January 2019.

#### Total services, daytime and out of hours

We combined all service codes encoding consultations (face to face, email, telephone, home visits) both from practice and out-of-hours (OOH) primary care.

#### Daytime services per 1000 listed patients

The number of all the different types of consultations provided during daytime per 1000 listed patients was retrieved.

#### Total services, out of hours

The sum of OOH consultations (face to face, telephone, and home visits) was calculated.

### Statistical analyses

The continuous variables on workload (hours, patient lists, and services) were categorised into relevant groups based on interpretation and distribution. The groups were an approximation of quartiles and were adjusted in size to enhance interpretation (for example, when a quartile cut-off fell in the middle of a cluster of datapoints, it would be moved slightly up or downwards, in order to group similar datapoints together). As described earlier, burnout was dichotomised (high or low) and estimates were analysed using a generalised linear model and a log-link, that is, binomial regression, yielding risk ratios (RRs) as the measure of the association between workload and burnout. All estimates are presented with 95% confidence intervals (CIs). We calculated the crude RRs and the adjusted RRs, using the lowest level of workload as reference, except for days in practice per week, where five was reference, and for OOH primary care, where the lowest level above zero was reference. We adjusted for the GP’s age (four categories), sex (two categories), and DADI score (quartiles). Analyses were conducted using Stata (version 17).

## Results

Descriptive data of the 312 included GPs are shown in Table 1. Of these, 72 (23%) had a high^9–12^ composite burnout score. Approximately half of GPs had between 1600 and 2000 listed patients, and 73% spent 5 days a week in practice. Among all included GPs, 61% did not have services registered for OOH primary care.

**Table 1.** Characteristics of the GPs in single-handed practices included in the study (*n* = 312)

GP characteristics	*n* (%)	
**Sex**		
Female	153 (49.0)	
Male	159 (51.0)	
**Age, years**		
<45	36 (11.5)	
45–54	92 (29.5)	
55–60	76 (24.4)	
≥60	108 (34.6)	
**Composite burnout score**		
3–4 (low)	17 (5.4)	
5–6 (low)	91 (29.2)	
7–8 (low)	132 (42.3)	
9–10 (high)	66 (21.2)	
11–12 (high)	6 (1.9)	
**Listed patients in 2019**		
<1600	81 (26.0)	
1600–1800	98 (31.4)	
1801–2000	70 (22.4)	
>2000	63 (20.2)	
**DADI score**		
≤24.8	79 (25.3)	
24.9–29.5	78 (25.0)	
29.6–36.0	81 (26.0)	
>36.0	74 (23.7)	
**Patient-related working hours in practice, daily**		
<7.5	40 (12.8)	
7.5–8.4	98 (31.4)	
8.5–9.4	86 (27.6)	
≥9.5	88 (28.2)	
**Days in practice per week**		
<5	62 (19.9)	
5	228 (73.1)	
>5	22 (7.1)	
**Total services, daytime and OOH**		
<9300	80 (25.6)	
9300–11 000	78 (25.0)	
11 001–13 000	80 (25.6)	
>13 000	74 (23.7)	
**Daytime services per 1000 listed patients**		
<5100	80 (25.6)	
5100–5900	76 (24.4)	
5901–6800	74 (23.7)	
>6800	82 (26.3)	
**Total services, OOH**		
0	189 (60.6)	
1–600	68 (21.8)	
>600	55 (17.6)	

OOH = out of hours

The crude and adjusted RRs are shown in Table 2. GPs spending >7.5 hours a day on patient-related tasks tended to have a higher probability of a high burnout score compared with GPs spending <7.5 hours a day on patient-related tasks. GPs spending 8.5–9.5 hours a day on patient-related tasks showed the highest RR, albeit not statistically significant (adjusted RR = 2.01, 95% CI = 0.90 to 4.51).

**Table 2.** Risk ratio (RR) of high burnout in relation to aspects of workload for single-handed GPs (*n* = 312)

High composite burnout score (9–12)					
	Unadjusted			Adjusted^a^	
*n* with high burnout (% of total)^b^		RR	95% CI	RR	95% CI
**Patient-related working hours in practice, daily**					
<7.5	6 (15.0)	ref		ref	
7.5–8.4	20 (20.4)	1.36	0.59 to 3.14	1.42	0.62 to 3.26
8.5–9.4	26 (30.2)	2.02	0.90 to 4.51	2.01	0.90 to 4.51
≥9.5	20 (22.7)	1.52	0.66 to 3.48	1.54	0.68 to 3.53
**Days in practice per week**					
<5	15 (24.2)	1.13	0.68 to 1.87	1.14	0.69 to 1.87
5	49 (21.5)	ref		ref	
>5	8 (36.4)	1.69	0.92 to 3.10	2.34	1.62 to 3.37
**Total services, daytime and OOH**					
<9300	20 (25.0)	ref		ref	
9300–11 000	15 (19.2)	0.77	0.43 to 1.39	0.72	0.40 to 1.27
11 001–13 000	18 (22.5)	0.9	0.52 to 1.57	0.85	0.49 to 1.45
>13 000	19 (25.7)	1.03	0.60 to 1.77	0.95	0.56 to 1.60
**Daytime services per 1000 listed patients**					
<5100	20 (25.0)	ref		ref	
5100–5900	14 (18.4)	0.74	0.40 to 1.35	0.75	0.41 to 1.36
5901–6800	19 (25.7)	1.03	0.60 to 1.77	1.08	0.63 to 1.83
>6800	19 (23.2)	0.93	0.54 to 1.60	0.89	0.53 to 1.50
**Total services, OOH**					
0	38 (20.1)	0.72	0.45 to 1.16	0.90	0.55 to 1.45
1–600	19 (27.9)	ref		ref	
>600	15 (27.3)	0.98	0.55 to 1.74	1.09	0.62 to 1.92

^a^Adjusted for age, sex, and DADI score. ^b^ *n* = number of burned-out GPs in each category, (%) = percentage of burned-out GPs of the total number of GPs in each category. E.g. Patient-related working hours in practice, daily, <7.5 hours, 6 GPs (15.0%) — 6 GPs with burnout in this group of 40 GPs in total in this group = 15%; 7.5–8.4 hours, 20 GPs (20.4%) — 20 GPs with burnout of 98 in total = 20.4%; 8.5–9.4 hours, 26 GPs (30.2%) — 26 GPs with burnout of 86 in total = 30.2%; ≥9.5 hours, 20 GPs (22.7%) — 20 GPs with burnout of 88 in total = 22.7%. OOH = out of hours

GPs working days per week in practice had a significantly higher risk of a high burnout score (adjusted RR = 2.34, 95% CI = 1.62 to 3.37).

There was no relation between number of registered services and risk of a high burnout score regarding daytime, OOH, nor when stratifying for number of listed patients.

## Discussion

### Summary

We investigated the association between burnout in Danish single-handed GPs and workload. We used data from a questionnaire survey from 2019 and the Danish health registers. We found that working >5 days a week in practice more than doubled the risk of a high burnout score compared with working 5 days a week. GPs are only obliged by contract to see patients 8 am to 4 pm, Monday to Friday. However, administrative work and so on may be postponed to the evenings and/or weekends. We do not know whether GPs working >5 days a week are motivated by, for instance, self-critical perfectionism,^38^ which increases risk of burnout, or by a burnout-induced reduction in workability, as suggested by Nørøxe *et al*.^34^ Both hypotheses could explain why we find more burned-out GPs in this group. Working longer hours than the average of the included GPs per day tended to be associated with increased risk of a high burnout score although not significantly. Regarding all variables on services provided, we saw no association to the risk of burnout.

### Strengths and limitations

The unique Danish registers gave access to precise and valid data on activity in the clinics. Further, the linkage of patients to clinics was based on the Danish personal identifier allocated to all Danes at birth. The National Health Service Registry is used to remunerate GPs, and has strength in its completeness.^35^ The identification of GPs through the PLO membership database minimised sampling bias. Compared with similar studies, the response rate was equivalent and satisfactory, especially considering the significant size of the questionnaire.

We could not collect register-based data on activities such as administration, research, and teaching. Nonetheless, the patient-related work accounts for the majority of a GP's workday,^1^ and therefore, supposedly, the majority of the workload.

We only examined working hours in practice, not in OOH primary care. GPs in different regions of Denmark have different contracts with the authorities, resulting in varying obligations regarding participation in OOH primary care. We asked the GPs only to report time spent on patient-related tasks. Therefore, the total amount of working hours will be higher than stated in this article.

By only including single-handed GPs, we aimed to minimise any differences in time spent on administrative tasks. Danish GPs do not carry out consultations with their patients during weekends. Hence, GPs reporting working beyond 5 days a week in practice may be a reflection of administrative tasks that have been postponed to the weekend.

Although self-report entails the risk of information bias, the self-reported data makes it possible for the GPs to register patient-related work that does not have a specific service code. Therefore, we found it relevant to analyse self-reported data alongside the register-based data regardless of the risk of information bias.

It was not possible to link each service to a specific GP, only to the practice. Thus, although the study was restricted to single-handed GPs without locums, we may still have counted contacts that were provided by other healthcare professionals (for example, nurses) in the practice. We only had access to data on number of employees, not how many hours they worked in practice nor the number of services they accounted for. Therefore, we chose not to include this as a variable in the study, since it would impart too much uncertainty to the results.

The restrictive inclusion criteria may limit the transferability of the findings to the entire GP population of Denmark. We do not know whether burned-out GPs hire locums to handle an overwhelming workload, or whether having a locum is protecting the GP against developing burnout. If this is the case, the association between workload and burnout may have been under as well as overestimated. However, these restrictions increase the validity of our analyses of the possible causal relationship between workload and burnout.

We used the MBI–HSS, rendering comparison with other studies possible. However, when more than one-third of all Danish GPs score high or moderately high on the MBI, but still have a high job satisfaction,^1^ one could argue that there might be a problem in the traditional categorical nature of the MBI. Therefore, we used the composite burnout score to approximate evaluating burnout as a linear construct.

### Comparison with existing literature

In contrast to earlier findings,^1–4^ we did not find an association between overall workload and burnout. This may be owing to differences in measures of workload. To our knowledge, this is the first study to compare register-based information on workload with GP burnout. Other studies have relied mainly on self-reported data regarding consultations, working hours, and patient lists.^2,3,12,15^ Our use of register-based data minimises risk of recall bias and the tendency to overestimate workload when experiencing burnout, providing us with more accurate data. This might explain why we do not rediscover the association other studies have found.

In this study, we included self-reported data on working hours as a measure to describe workload. We found stronger associations between self-reported data and burnout than between register-based data and burnout. In 2015, Picquendar *et al* performed a cross-sectional survey on burnout and medical shortage of 501 French GPs.^13^ Their results suggested that it was the qualitative workload rather than the quantitative workload that drove burnout development. Whether the predictive value of the qualitative workload is better than the predictive value of the quantitative workload concerning burnout is difficult to determine based on the available studies. Register-based data has several limitations. First, register-based data only reflect number of tasks with a service code and not content of the service. Second, the appropriate time window for collecting register data, which are not yet influenced by a rise in burnout and still reflects a relevant exposure for the present burnout status, has not been established. Perceived workload is not without flaws either. First, in cross-sectional studies it is difficult to separate the perceived burden of the workload from the burnout status. Second, factors that may predispose to burnout may also be associated with increased perceived workload; for example, personality traits,^38^ and poor-working environment and workflow among practice staff.^39^ Taken together, the results have shown that the workload, as measured by service registers, does not relate to the perceived workload and the burnout status. A reasonable explanation of our findings is that the experienced workload is true and represents the GP’s feeling of the link between wellbeing at work and the number of services provided.

In a two-wave study, Pedersen and Vedsted^32^ compared composite burnout scores of Danish GPs from 2016 and 2019, and found that GPs presenting with a high composite burnout score in 2016 were 12 times as likely to have a high burnout score in 2019, making it by far the strongest predictor of burnout among all factors studied. Thus, a large quantity of GPs with burnout have likely had it long before the data collection took place, and therefore, we do not know if they have decreased their working hours after developing burnout symptoms, masking a possible relation between workload and burnout.

A study from 2016 analysed the non-responders of a similar report and found that caring for a higher number of older, comorbid, or deprived patients was associated with non-response,^40^ implying that GPs facing greater demands at work are less likely to respond to surveys. Reasonably, this also applies to our data, which may leave us to underestimate the association between workload and burnout.

### Implications for research

Understanding the causes leading to the increase in GP burnout is important in maintaining and recruiting a sustainable GP workforce, hereby strengthening the entire healthcare system. In this study, workload in itself was not associated with burnout. Other factors than workload may be responsible for burnout. Further exploration on personal and environmental factors related to positive and negative effects on burnout is suggested. Provision of registers linking healthcare services to a specific GP and not only to a practice, would make it possible to repeat this study in a broader and more representative GP population.

All data for this article were collected before the COVID-19 pandemic, which therefore has no effect on the results. As burnout rates have increased post-COVID, the level of burnout identified in this article may underestimate the current level.
